# Technology Use and Monitoring Preferences in Older Adults: Implications for Passive Remote Monitoring

**DOI:** 10.1093/geroni/igaf122.2858

**Published:** 2025-12-31

**Authors:** Molly Lawrence, Sarah Prieto, Alexa Vasquez, Alyssa De Vito

**Affiliations:** Alpert Medical School at Brown University, Providence, Rhode Island, United States; Brown University, Providence, Rhode Island, United States; Brown University, Providence, Rhode Island, United States; Brown University, Providence, Rhode Island, United States

## Abstract

Rising Alzheimer’s disease (AD) rates necessitate better detection of early behavioral changes. Passive remote monitoring (PRM), technology using sensor-based data to monitor behavior, can describe a person’s day-to-day functioning. Acceptability of PRM among older adults remains uncertain. We investigated older adults’ use of technology and their openness to monitoring of technology use using a 25-30-minute online survey regarding current technology use and openness to PRM of technology use. Participants were 171 older adults with normal cognition or mild cognitive impairment (mean age=56, 66% Female, 29% White, 28% Black/African American, 18% Latinx, 22% Asian, 3% Mixed/Other Race) .Most respondents reported using smartphones for: texting (95.9%), calls (95.3%), camera/photos (94.2%) and e-mail (90.6%). Fewer respondents reported using phones for games (60.2%), health monitoring (60.2%) or digital assistance (38%). Females used GPS more than males (p < .05). Young-old adults (age < 55) used applications for organization, digital assistance, e-mail, social media, and health tools more than Old-older adults. Most of the sample reported openness to monitoring of: mobility (86%), overall time spent on phone (80.1%) and speed/errors in typing (76.6%). Respondents were less open to PRM of app use type (69%), GPS location (63.8%) and number of incoming/outgoing calls (61.9%). No significant differences emerged regarding openness to monitoring based on age or gender (p>.05). These results demonstrate that older adults use technology for a range of daily tasks, indicating that PRM may be acceptable in this population. Older adults also may be willing to have some tasks monitored to detect cognitive impairment.

